# Comparing technology and regulatory landscape of probiotics as food, dietary supplements and live biotherapeutics

**DOI:** 10.3389/fmicb.2023.1272754

**Published:** 2023-12-19

**Authors:** Irina Spacova, Sylvie Binda, Jessica Anne ter Haar, Solange Henoud, Sophie Legrain-Raspaud, James Dekker, Jordi Espadaler-Mazo, Philippe Langella, Rebeca Martín, Marco Pane, Arthur C. Ouwehand

**Affiliations:** ^1^Research Group Environmental Ecology and Applied Microbiology, Department of Bioscience Engineering, University of Antwerp, Antwerp, Belgium; ^2^Rosell Institute for Microbiome and Probiotics, Montreal, QC, Canada; ^3^International Probiotics Association, Los Angeles, CA, United States; ^4^Gnosis by Lesaffre, Marcq-en-Baroeul, France; ^5^Fonterra Research and Development Centre Co., Ltd., Palmerston North, New Zealand; ^6^Department of R&D, AB-Biotics SA (Kaneka Group), Barcelona, Spain; ^7^Université Paris-Saclay, INRAE, AgroParisTech, Micalis Institute, Jouy-en-Josas, France; ^8^Probiotical Research, Novara, Italy; ^9^Global Health and Nutrition Science, IFF Health, Kantvik, Finland

**Keywords:** probiotic, supplement, drug, regulation, pharmabiotics, manufacturing, clinical trials, product development

## Abstract

Application of beneficial microorganisms as probiotics targets a broad range of intended uses, from maintaining health and supporting normal bodily functions to curing and preventing diseases. Currently, three main regulatory fields of probiotic products can be defined depending on their intended use: the more similar probiotic foods and probiotic dietary supplements, and live biotherapeutic products. However, it is not always straightforward to classify a probiotic product into one of these categories. The regulatory nuances of developing, manufacturing, investigating and applying each category of probiotic products are not universal, and not always apparent to those unfamiliar with the various global probiotic regulatory guidelines. Various global markets can be significantly different regarding legislation, possible claims, market value and quality requirements for the development and commercialization of probiotic products. Furthermore, different probiotic product categories are also linked with variable costs at different stages of product development. This review outlines the current landscape comparing probiotic foods, probiotic dietary supplements, and live biotherapeutics as probiotic products from a regulatory lens, focusing on product development, manufacturing and production, and clinical research agenda. The aim is to inform and promote a better understanding among stakeholders by outlining the expectations and performance for each probiotic product category, depending on their intended use and targeted geographical region.

## 1 Introduction

Soon after the discovery that certain microbes may cause disease came the realization that specific microbes also have the potential to promote health. This concept was most notably put forward by Henry Tissier in 1900 in his work “Recherches sur la flore intestinale des nourrissons (état normal et pathologique),” and by Ilya Metchnikov in 1907 in his publication “Etudes sur la nature humaine: essai de philosophie optimiste.” This idea of beneficial microbes has over time entered different applications: first and foremost, in (functional) foods, but subsequently also in dietary supplements, pharmaceuticals, cosmetics, self-care and natural health products, to name only a few application areas. The microbes in these application areas can generally be captured within the term “probiotic,” “live microorganisms that, when administered in adequate amounts, confer a health benefit on the host” (Hill et al., 2014). The different applications and intended uses require different approaches as to how they are studied, for what endpoints, who they target, how they are to be promoted, and the resulting applicable regulatory requirements.

The continuum from maintaining health and supporting normal bodily functions to curing and preventing diseases is well-defined in food and drug regulations, though the nuances are not always apparent to those unfamiliar with the various global probiotic regulatory guidelines. This makes it challenging at times to delineate health benefits intended for food or dietary supplements from those intended for drugs. Within the current perceptions of probiotics, several misconceptions, and by extension unreasonable expectations exist regarding probiotics within their three main regulatory fields of application: probiotic food (PF), probiotic dietary supplement (PDS), or live biotherapeutic products (LBPs). Here, PF and PDS are, respectively defined as food products and supplements containing living microorganisms according to the current probiotic definition (Hill et al., 2014) that are “intended to maintain or enhance a healthy state in a healthy or at-risk population” (Cordaillat-Simmons et al., 2020). Consequently, fermented foods do not necessarily fall under PF, unless they contain live microbes characterized to the strain level and have been documented to provide a health benefit (Marco et al., 2021). Furthermore, in this article, we define LBPs as pharmaceutical/medicinal products that consist of “a biological product that: (1) contains live organisms, such as bacteria or yeasts; (2) is applicable to the prevention, treatment, or cure of a disease or condition in human beings; and (3) is not a vaccine” (United States Food and Drug Administration [FDA], 2016; The European Directorate for the Quality of Medicines and HealthCare [EDQM], 2019). This definition is in line with the historical concept of “pharmabiotics” (Cordaillat-Simmons et al., 2020).

The different probiotic categories (PF, PDS, LBPs) and their corresponding regulation therefore depend on the product’s intended use and whether this intention is to maintain health or to prevent or cure a disease or its symptoms. PF such as probiotic yogurts and PDS such as probiotic capsules might not always require a lengthy regulatory process before they are marketed and are regulated in a regional/country specific regulation. PF and PDS generally target healthy populations, for disease risk reduction or dietary management of a disease and claiming benefits is subject to variable regulatory criteria depending on the jurisdiction. Notwithstanding this, probiotics have been shown to benefit both healthy populations, reducing risk for upper respiratory tract infections (Hao et al., 2015), and patients, reducing risk for antibiotics associated diarrhea (Goodman et al., 2021) and as adjunct to *Helicobacter pylori* eradication (Wang et al., 2023b) or reducing risk for necrotizing enterocolitis (Wang et al., 2023a). On another hand, the regulatory process for a probiotic product as an LBP to treat, cure or prevent diseases remains more consistently challenging across the globe. This is because traditional drug regulations are not well-suited for living microorganisms due to the complexity within the nature of a microbial entity. Further, their multifactorial modes of action, and strict criteria related to clinical safety, efficacy and quality that must be met to obtain marketing authorization by competent authorities.

**FIGURE 1:**
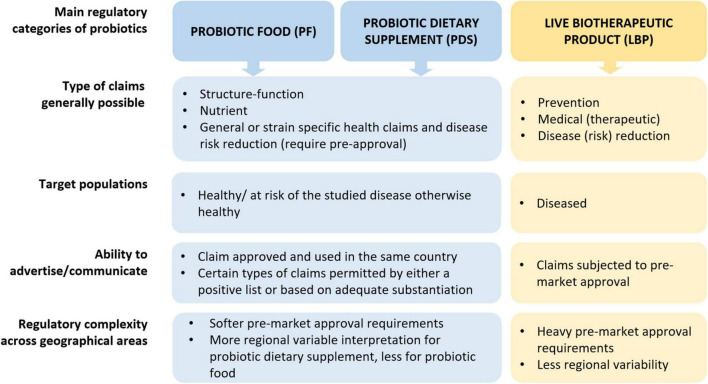
Overview of main regulatory categories of probiotics (PF, PDS, LBPs). Of note, the use of PF and PDS is not excluded in a diseased individual to support health for similar purposes as in healthy individuals, however here we emphasize the target population from a regulatory perspective. Elaborated by the authors from various sources.

The varied technical and regulatory challenges in various global markets between PF, PDS and LBPs may mean that a company needs to satisfy multiple sets of differing quality requirements to commercialize the same product or organism. Consequently, the feasibility of developing a new probiotic product is not universal, as significant differences exist between countries regarding legislation, possible claims, market value, etc. (Figure 1; Table 1 column “Zone impact”). Variable costs at different stages of product development may likewise hinder the emergence of new probiotic products depending on the PF, PDS or LBPs category (Figure 1; Table 1). While developing a probiotic strain as an LBP might offer broader possibilities to protect intellectual property, as well as relatively high sector revenue and possibly product margins, the current cost to bring such a product to the market is rather similar to drugs and thus considerably higher compared to PDS and PFs. Clinical trials to substantiate beneficial effects of LBPs are typically more stringent compared to PFs or PDS as they require longer duration, must assess the benefit-risk balance with the goal of curing/mitigating a disease (Table 2). On the other hand, LBPs focus on specific well-defined health/clinical endpoints and biomarkers. In the case of PF or PDS, the clinical studies focus on maintaining health, supporting normal bodily functions, reducing risk for a condition or reducing specific disease factors in a healthy population; here, the expected efficacy can be modest. The requirement to perform clinical trials with “diseased” compared to “healthy” populations to show efficacy, or to identify clinical endpoints in healthy populations not characterized by clinical symptoms, can often stymy research contributions to regulatory dossiers (EFSA Panel on Dietetic Products, Nutrition and Allergies, 2021). Nevertheless, such challenges may also stimulate creativity and catalyze new approaches (Zeilstra et al., 2018; Yadav and Shukla, 2019; Cunningham et al., 2021; Trukhachev et al., 2021).

**TABLE 1:** Hypothetical comparison, based on expert opinion, of the development costs and return for a probiotic food (PF), probiotic dietary supplements (PDS), medical foods/foods for specific medical purposes (MF/FSMP), and live biotherapeutics (LBPs) probiotic products.

Comparative parameters	PF	PDS	MF/FSMP	LBPs	“Zone” impact*
Reimbursement schemes	No	No	Same as other MF/FSMP	Yes, same as other drugs	
Average R&D cost	Low-Medium	Low-Medium	Medium-high	Medium-high	
Available R&D funding	Limited	Limited	Limited	Medium	
Patenting possibilities	Limited	Limited	Yes	Yes	
Manufacturing costs	Medium-high	Medium-high	High	High	
Extent of regulatory requirements	Low	Low-Medium	Medium-High	High	
Market size	Broad	Broad	Broad	Broad	
Product revenue	Low	Low	Medium	High	
Cost to bring to market	Medium	Medium	Medium	High	
Total value of markets	Low	Low	Medium	Medium-High	
Return on investment	Low	Low	Low-medium	Medium	

The levels indicated assume that PF and PDS are indeed foods and supplements and as such should be affordable and cannot claim effects on disease. MF and FSMP are also foods but serve a specific medical purpose that needs to be documented and can be marketed at a somewhat higher price than their regular counterparts. LBP’s on the other hand are drugs that require thorough documentation and can be sold at drug prices. These comparisons between the product categories are intended to give an indication of hierarchy/rank/weight on the various elements, not as an empirical grading system. Many of these elements would be notoriously difficult to objectively quantify which is not within the scope of this article. *Column “zone impact”: the system of the traffic lights means that there are significant differences for, e.g., health claims, reimbursement schemes, etc., according to the jurisdiction where one operates. Red indicates that there are differences that will most likely influence the final cost. Orange indicates that there may exist influence on the final cost. Green indicates there are little or no influence on the final costs expected.

**TABLE 2:** Comparison of considerations in designing, executing, and interpreting a clinical trial for probiotic food (PF), probiotic dietary supplements (PDS), medical foods/foods for specific medical purposes (MF/FSMP) and live biotherapeutics (LBPs) in oral applications.

	Trial type				Commonalities to note
	PF	PDS	MF/FSMP	LBPs	
Acceptance of side effects	Very low	Very low	Very low	Yes	Managing informed consent
Tolerability of product	Good, but sometimes gastrointestinal discomfort is noted	Good, but sometimes gastrointestinal discomfort is noted	Good, but sometimes gastrointestinal discomfort is noted	Expected to be good, albeit exceptions can occur	Have a system in place to record adverse events
Incidence of side effects	Very low	Very low	Very low	Low	Have a system in place to record adverse events
Severity of side effects	Low to very high	Low to very high	Low to very high	Low to very high	
Average severity of AEs or SAEs	Minimal	Minimal	Minimal	Minimal	
Reporting of adverse events	Variable (but generally required)	Variable (but generally required)	Variable (but generally required)	Extensive and required	A requirement for all types of clinical studies
Expected efficacy	Modest	Modest	Major	Major	None
Actual efficacy (responders)	Modest	Modest	Modest-major	Modest-major	None
Response to efficacy	Variable	Variable	Variable to consistent	Variable to consistent	None
Target population	Typically healthy/at risk of a disease	Typically healthy/at risk of a disease	Typically diseased	Typically diseased	None
Study population	Heterogeneous	Heterogeneous	Relatively homogenous	Relatively homogenous	None
Preclinical data/baseline pilot data	Variable	Variable	Extensive	Extensive	None
What product is being studied in clinical trial?*	Not always finished product	Not always finished product	Finished product	Finished product	None
Size of study population**	Small/variable	Small/variable	Variable	Variable	Do power calculation
Study duration/follow-up**	Short/minimal	Short/minimal	Short/minimal	Variable	None
Trial registration/Data deposition	Yes/inconsistent	Yes/inconsistent	Yes/yes	Yes/yes	Required
Reporting results	Inconsistent	Inconsistent	Yes	Yes	Should be a requirement
Reproducibility	Variable	Variable	Variable	Variable	Required by some authorities
Statistical significance	Variable	Variable	Variable	Variable	Statistical plan should be developed and ‘locked’ before intervention start and completion
Health endpoint	Maintaining health, supporting normal bodily functions, reducing risk for a condition, reducing specific disease factors/biomarkers	Maintaining health, supporting normal bodily functions, reducing risk for a condition, reducing specific disease factors/biomarkers	Curing/mitigating disease	Curing/mitigating disease	Define upfront what is the primary outcome
Established health endpoint	No/not always	No/not always	Yes	Yes	None
Systemic function	Complex networks	Complex networks	Complex networks	Complex networks	None
Known mechanism of action	Not always known	Not always known	Generally better established than PF/PDS	Generally better established than PF/PDS	R&D challenges to justify and investigate the hypothesis within the available research budget
Biomarkers	Minimal established, more surrogates	Minimal established, more surrogates	Typically well established	Typically well established	None
Quality control	Dosing live microorganisms	Dosing live microorganisms	Dosing live microorganisms	Dosing live microorganisms	Live microbe counts
Administration	Non-systemic	Non-systemic	Non-systemic	Non-systemic	None

The levels indicated assume that PF and PDS are indeed foods and supplements and as such should pose no risk to the general consumer and cannot claim effects on disease. MF and FSMP are also foods but are consumed by a specific patient population, the benefits in this population need to be documented. LBP’s are drugs that target a specific patient population, this requires thorough documentation. As the effect of LBP’s is expected to be bigger, side effects are also easier accepted. These comparisons between the product categories are intended to give an indication of hierarchy/rank/weight on the various elements, not as an empirical grading system. Many of these elements would be notoriously difficult to objectively quantify which is not within the scope of this article. *depends on product development plan/target product profile. **depends on the phase of randomized controlled trial/type of study, but in pharma for instance, minimal weight management trial duration is 6 months.

The purpose and intent of this review is to discuss and help to promote a better understanding regarding what can be expected from PF and PDS versus LBPs in terms of finished products. The intended use and thereby the role of regulations as a focus lens will frame the context of product development, manufacturing and production, and the clinical research agenda.

Of note, the focus of this article is on oral applications of PF, PDS, and LBPs, therefore medical devices for targeted probiotic application to non-gut body sites (e.g., nose sprays and vagitories) and topical cosmetic products containing probiotics will not be discussed in detail. Next generation probiotics (NGP), which would be Novel Foods (EU) or New Dietary Ingredients (USA) are not specifically considered. Their intended use would most often coincide with LBPs. Furthermore, although the concept of PF, PDS and LBPs can be extended to (companion) animals, this falls outside the scope of this review and will not be addressed. Similarly, other “biotics” such as postbiotics fall beyond the purview of this review, partly due to space limitations, as well as the ongoing controversies surrounding their precise definition (Aguilar-Toala et al., 2021). While we discuss the development and regulatory processes until a product reaches the market, the specific content of claims and marketing strategies are also outside the scope of this review.

## 2 Regulations

Probiotics are regulated depending on their intended use, which dictates the resulting regulatory category (PF, PDS, or LBPs). The intended use is defined by the totality of the answers to all the following questions (Figure 1):

• Where (country, jurisdiction).
• What (ingredient, blend of ingredients, a capsule, a cereal bar, a liquid, etc.).
• Why (to support or maintain normal bodily functions, to reduce the risk of a disease, to prevent, cure or mitigate a disease/condition or its symptoms).

Regulations, and subsequent requirements, as well as pre-market or post-market oversight from authorities vary and are country/region and category specific. In most countries, probiotics are sold either under food or drug regulations. However, very few countries have developed specific regulations for probiotics within a specific category dedicated for supplements which outlines clear expectations on characterization, safety, efficacy and quality. PF and PDS generally fall under food law, with or without a specific framework such as “Foods with probiotics” (Argentina), “Food products with additions” (Saudi Arabia, Mexico, Morocco), “Foods with function claims” (Japan), “Health functional food” (South Korea), or “Dietary supplements” (USA). Of note, probiotic products can be categorized as drugs due to their formulations or health claims, but also because specific PDS would be regulated under drug law in countries such as South Africa and Australia (Paraskevakos, 2020).

Many countries also have positive lists of specific organisms, at species or strain level. The microorganisms on such lists may be sold in a precise jurisdiction, under pre-established safety conditions without the need of further pre-marketing approvals from the authorities unless the intended use shifts away from the boundaries imparted by the list, whether explicitly or implicitly. The qualified presumption of safety (QPS) list regulates microorganisms intentionally introduced into the food and feed chain in the European Union (EU) (EFSA BIOHAZ Panel et al., 2022), including in PF and PDS. Similarly, the United States Food and Drug Administration (FDA) can, upon request, evaluate if a food ingredient (e.g., a microbial strain) can be considered Generally Recognized as Safe (GRAS), based on a dossier submitted. When the FDA ‘has no questions’ the ingredient is considered safe under the conditions of its intended use. Importantly, the GRAS status is very specific concerning the microbial strain, dose and its uses (Mattia and Merker, 2008). When probiotic products are intended to directly or indirectly treat, prevent, cure, mitigate a disease and/or a condition, probiotic products are drugs and must undergo a long and laborious pre-marketing approval process (relevant examples for LBPs in different geographies are listed in Supplementary Table 1). For some food supplement categories in countries like Canada (Natural Health Products) and Brazil (probiotic supplements) a pre-marketing approval is required. However, companies are permitted to conduct clinical trials and make claims that are intended to support normal functions, correct certain functions or reduce the risks of certain diseases.

In cases where pre-marketing approval of food and supplements is not required *per se*, like the United States of America and sometimes Canada, generally any information used to represent the product should be truthful, non-misleading and adequately substantiated. Communication goes beyond just product labels and advertising, to cover company websites and testimonials which are subject to enforcement action and penalties; there are clear regulations surrounding these restrictions. Countries like Canada have established possible generic claims with pre-determined wording while others permit the use of customized statements - provided they are reflective of the supportive data for structure/function or content claims. While other countries do not allow any claims for PF/PDS, or specifically prohibit them for probiotics, while allowing access to market based on meeting quality and safety requirements, and or based on a positive list.

In Europe access to market for PF/PDS can be quick and straightforward based on the QPS list mentioned above. However, while many health claims are allowed in the EU for other foods/supplements, the European Commission guidelines (European Commission [EC], 2007) prohibited the use of the term “probiotic” on PDS labels. This has formed a distinct barrier for probiotic marketing in the EU compared to other regional markets. Nevertheless, the situation has continued to develop and is not currently harmonized across the EU market, as several EU member states (e.g., Bulgaria, Cyprus, Czechia, Denmark, France, Greece, Italy, Malta, the Netherlands, Poland, and Spain) have taken different approaches to enable using the term “probiotic” or “contains probiotic” on food products as long as this does not constitute a health claim (IPA Europe, 2023).

Access to market is fairly easy for PF and PDS in the EU, as long as they contain microorganisms that are included in a positive list. The regulatory framework provides provisions to allow specific health claims, and the European Food Safety Authority (EFSA) has established clear and useful guidance on how to study and make claims for probiotics in different indications where benefits can be extrapolated to the general population. Notwithstanding this, the approval of health claims for probiotics has remained highly challenging in the EU. This is predominantly due to uncertainty of the assessment by EFSA linked to the required evidence to establish cause and effect relationship between the consumption of the probiotic and the beneficial physiological effect. More than 400 health claim applications have been submitted in the EU and were rejected. Yet, under the same overall regulation in a different article, a probiotic health claim has been approved by EFSA related to improved lactose digestion in consumers with lactose maldigestion by yoghurt containing at least 10^8^ CFU/ml *Lactobacillus delbrueckii* subsp. *bulgaricus* and *Streptococcus thermophilus* (EFSA Panel on Dietetic Products, Nutrition and Allergies, 2010).

In Canada probiotics can fall under the category of foods or natural health products (NHPs). NHPs are a special regulatory category distinct from Foods, regulated by a different directorate of Health Canada, the Natural and Non-Prescription Products Directorate, who established specific considerations for probiotics. This category is aligned with scientific advancements and allows probiotics to be appropriately studied and to translate positive outcomes into specific claims through an evaluation process of 7 months. The level of required evidence depends on the proposed claim. NHPs can also make pre-established general claims and access the market within 60 days when they fully meet the conditions outlined in the probiotics monograph (Health Canada, 2023). NHPs cannot be sold in Canada without being approved and without making claims. As NHPs, probiotics can thus make a wide range of claims from maintenance-promotion of health to reduction of risk, management of symptoms or adjunctive treatments. However, the status is very different for PFs and there is currently no clearly defined category for LBPs in Canada as compared to Europe or the USA.

In the USA, PDS fall under the dietary supplement category which has special considerations but remains under the food umbrella. Probiotics, like any other substances, fall under the requirements and provisions of the Dietary Supplement Health and Education Act (DSHEA, 1994). Probiotics, whether PF or PDS, cannot make or imply by any representation, claims to treat, cure, prevent or mitigate a disease, a condition or any of their symptoms. The line between disease and healthy is very fine, making it practically very challenging to make and substantiate claims in the USA in a lawful manner, while DSHEA enabled access to the market making structure function claims provided they are truthful, not misleading and substantiated by adequate reliable substantiation. The regulations are complicated and very different across the border in Canada, where probiotic PDS can make approved claims, and in Mexico, where PDS cannot make any claims at all. Furthermore in the USA, clinical trial guidelines were designed for disease endpoints, which adds to the complexity of how to appropriately study and be able to promote the outcomes of the studies in PDS and PF. Regarding LBPs in the USA, the FDA has recently approved VOWST (live-brpk, formerly SER-109) with fecal bacterial spores from healthy volunteers against *Clostridioides difficile* infection for medical use (Supplementary Table 1; Khanna et al., 2022).

In addition to the three categories described above, there are also medical foods (MF) or foods for specific medical purposes (FSMPs) in which probiotics can also fit. The former is a category that has existed with a statutory definition since the amendment of the Orphan Drug Act in 1988 (United States Code, 2001) in the USA and concerns foods which are specifically formulated to be consumed or administered enterally under the supervision of a physician and which are intended for the dietary management of a disease or condition for which distinctive nutritional requirements, based on recognized scientific principles, are established by medical evaluation (United States Food and Drug Administration [FDA], 2023). Similarly, EFSA defines FSMPs as a category of foods for particular nutritional uses specially processed or formulated and intended for the dietary management of patients and to be used under medical supervision (EFSA, 2021).

The above regulations are just a small sample of the regulatory complexity, highlighting there is no international harmonized standard for how to deal with probiotic regulations, not only across regions but also within the same country, depending on whether they are PFs, PDS, or LBPs. While one may argue the pros and cons of the current regulatory environment for PF or PDS worldwide, it is certainly correct to claim that they are, like LBPs, subject to different regulatory provisions, depending on their intended use. For more detailed discussions on the regulation concerning probiotics, the reader is referred to: (Paraskevakos, 2020; Chieffi et al., 2022).

## 3 Manufacturing and production

To ensure appropriate, effective, and reproducible application of the probiotic product in the intended regulatory category, well-controlled manufacturing processes and quality assurance procedures are required. We will not discuss here the intrinsic safety of specific probiotic strains, as many strains belong to species with a long history of safe use in foods (Gueimonde et al., 2004). This forms the basis of the QPS concept. Here, we will focus on quality and safety aspects for finished probiotic products. For details on appropriate testing of strains belonging to other species, the reader is referred to, e.g. (Roe et al., 2022).

There are specific quality and safety considerations for PF, PDS, and LBPs related to the finished products. For example, for quality management in drug regulations, notions of DS (Drug Substance) and DP (Drug Products) with different guidelines must be applied for final quality assessment, which are not applicable to PF and PDS. This is related to the intended use and the target population as discussed in the section on the role of the regulation, above, as well as in a recent publication discussing testing requirements for different probiotic product types and target populations (Merenstein et al., 2023). This also dictates how probiotics for these different categories are produced, though there is significant overlap in the industrial production process for all categories.

The manufacturing of single strain PF, PDS, and LBPs starts with a culture seed stock, which has been carefully prepared to contain (usually) a single pure strain and verified to be free of contaminants by quality control (QC) testing. This stock is used in a limited number of sequential seed fermentations to achieve the desired biomass to ultimately inoculate the main fermentation vessel. The rationale behind this procedure is to limit the number of generations from seed stock to product, thereby reducing any potential risk for genetic drift and cross-contamination. Products containing a mixture of strains are commonly fermented as single strains as described above, and the resulting products are subsequently formulated in their desired ratio.

Most probiotic microorganisms are fastidious in terms of nutritional requirements for growth and performance and require complex culture media. The transfer from laboratory conditions to industrial conditions can eventually impact the applicability of the beneficial probiotic strain if it cannot be industrialized with sufficient biomass to be formulated into a commercial product. The raw materials for the industrial-scale media need to be carefully selected and controlled, and their quality of use should be ascertained ahead of time. This is important for microbes that require specific growth conditions and/or strict anaerobic atmospheres. Therefore, it is important to understand the organisms’ auxotrophic requirements and their global bacterial physiology. In an effective translational approach, the industrialization of a probiotic strain should be assessed in parallel with its functionality. In some cases, compromise is warranted to balance these two important aspects in a manufacturing setting. Furthermore, additional information should be known and approved by the quality control team before use, including GMO status; allergen status; the raw material purchasing specifications, including chemical, physical, and microbiological; ingredient quality graded for the appropriate category; pesticides; irradiation; Kosher/Halal rating; storage condition and shelf life (Fenster et al., 2019).

Hazard Analysis and Critical Control Points (HACCP) is a system designed to identify, evaluate, and establish acceptable protocols to minimize food safety hazards from biological, chemical, and physical hazards in the production process, and it is relevant in the manufacture of probiotics. It is a system that focuses on the prevention of food safety problems rather than relying on end-product testing but is applicable to other production processes as well. HACCP plans must be developed by the food processor to identify critical control points in the production process. Then, monitoring and controlling measures must be established to ensure that these critical control points are checked (Food Standards Agency, 2017; Markel Services Inc, 2023). Although HACCP has become an internationally recognized system that is used in food production and handling facilities worldwide, there is no globally accepted 3rd party certification program. However, organizations such as the International Probiotics Association (IPA) and International Organization for Standardization (ISO) have their attention on developing such programs.

Current Good Manufacturing practices (cGMP) are guidelines provided by the FDA for the production and quality control of drugs (United States Food and Drug Administration [FDA], 2021; CFI Team, 2023). These guidelines ensure that all products meet the same high standards of quality, safety and efficacy, regardless of their category. cGMPs provide for systems that assure proper design, monitoring, and control of manufacturing processes and facilities. This includes establishing and following written procedures and documentation, using properly trained personnel, verifying and validating processes, and maintaining proper records. cGMPs are enforced by the FDA to ensure that all products are safe and effective for their intended uses, including LBPs and MF (United States Food and Drug Administration [FDA], 2023). FSMPs, however, can be manufactured under GMP standards (EFSA, 2021).

As PF, PDS, and LBPs are all intended to contain live microorganisms, the risk of microbial contaminants can be higher compared to traditional drug products subjected to intentional sterilization. Due to differences in target populations, the microbial quality control required for PF and PDS versus LBP is an important consideration. LBPs target vulnerable and sick populations, therefore more stringent product quality control is required compared to PF and PDS targeting such populations, regarding for potential microbiological contaminants, such as *Listeria monocytogenes* and *Bacillus cereus* (Merenstein et al., 2023). Furthermore, manufacturing live microorganisms in PF, PDS, and LBPs can result in higher batch-to-batch variation compared to traditional chemically manufactured drugs (Cordaillat-Simmons et al., 2020), and upscaling/manufacturing may alter certain probiotic strain characteristics previously observed in laboratory conditions (Nivoliez et al., 2015). Thus, efficient parametric, attribute, and procedural controls during manufacturing are required to retain reproducible health-promoting and/or disease-mitigating effects, with special attention to parameters that may influence the probiotic mode-of-action (Cordaillat-Simmons et al., 2020). This is especially true for the LBPs category that has more stringent requirements set by the drug authorities regarding product quality, safety and efficacy.

## 4 Product development

Significant differences exist in the extent of regulatory requirements between PF, PDS and LBPs (Figure 1), which can affect the overall process and costs. Therefore, it is important for companies to establish the intended use and thus the regulatory status of the probiotic product during the early stages of the product development. To demonstrate health benefits and/or disease-mitigating effects, all three categories of probiotic products typically undergo a preclinical research stage before moving to clinical trials.

The product development of PF, PDS, and LBPs can differ substantially depending on the intended use and thus the regulatory framework, which dictates the necessary product development requirements. This should already be considered during preclinical planning and taken into consideration in ensuing clinical trials. Discovery research and preclinical investigations are performed before initiating clinical trials, potentially including *in silico, in vitro*, *ex vivo* and *in vivo* testing. Preclinical testing of a probiotic strain can support and inform its intended use in the target population and may help in the development of the metabolic maps. For all probiotic categories, sufficient characterization of the strain is required, starting from strain identification, and taxonomic assignment according to most recent nomenclature, e.g., (Zheng et al., 2020) for lactobacilli to which many commonly used probiotics belong to. Use of strains that pose minimal or no risk (assessed through documented history of safe use, whole genome sequencing and bioinformatics, and other experimental techniques) is likewise a prerequisite for any probiotic strain or blend, and ultimately product; however, the risk analysis requirements can differ between PF/PDS and LBPs categories. Generally, more extensive preclinical data is required for LBPs compared to PF/PDS (Table 2). For example, PF/PDS with a documented history of safe use testing in animal models may not be necessary, as these products can be applied directly in the intended healthy populations – with additional medical observation advised in vulnerable groups (Sanders et al., 2016). On the other hand, a more extensive general toxicity/safety assessment based on a combination of *in vitro*, *ex vivo*, and possible experiments in animal models is advised for LBPs which is more like traditional drugs (Rouanet et al., 2020). Of note, multiple organizations have long lobbied to reduce the use of animal models where possible (see section on Clinical research strategies). Recently the FDA can promote a drug or biologic to human trials after either animal or non-animal tests (Wadman, 2023).

In preclinical research, many of the discovery pipelines for identification of beneficial microbes and their concomitant health-mediating mechanisms entail growing and characterizing them under laboratory conditions. These conditions can be different from industrial ones, which might impact the final product’s efficacy, especially when the effector depends on the growth conditions. In addition, the probiotic strains may interact with additives and matrices used in their final formulation and affect their efficacy as well. It is therefore recommended to work with research material that is produced under commercial production conditions, whenever possible. Whatever the final formulation, lyophilizate or fermented product, the downstream process will have to maintain and protect the biomass integrity and activity. If the functionality is linked to specific surface component (like pili or surface proteins, or the presence of certain enzymes), it is essential to track the impact of the downstream processing to allow for maximum retention of the desired functionalities (Lebeer et al., 2018).

Therefore, if the structure and function of a certain probiotic health effector is known and linked to biological performance, these parameters should be monitored throughout the development flowchart to ensure their preservation. Establishing a metabolic map with some of the key metabolites produced can also help to model the strain and product development processes and their potential impact on efficacy, as mentioned in Box 1, which is applicable to all three probiotic product categories. Preferably, the production process should be managed to the extent that the steps that can impact the effector are known and controlled. In other words, it is important to develop a production process mapping that identifies the degree of flexibility that the process can have, and the steps where changes can occur without damaging the expected output. With this perspective in mind, identifying the thresholds between minor and major changes in the processes is critical for companies.

BOX 1 **Key points to be considered for an efficacious product development with probiotic microbes applicable to all three probiotic product categories discussed here.**

1. Characterize the probiotic strain(s) included in the probiotic product

- To demonstrate probiotic safety (level of taxonomic depth depends on the regulatory category)
- To demonstrate probiotic efficacy (level and type of evidence depends on the regulatory category)
- To identify metabolic activities and/or structures demonstrated to be of importance for efficacy
- To conserve these key metabolic activities and/or structures throughout production
- To maintain probiotic viability and activity

2. Define the product

- To choose the final probiotic form to be used
- To choose the potential matrix (functional or inert)
- To assess stability
- To choose the possible additives to have a better stability
- To demonstrate the safety, quality, and efficacy of the product

Preferably, the product is tested in its final delivery matrix in preclinical tests that have enabled the choice and characterization of the beneficial microorganism. In line with the above point, the optimal situation is when the effector is known and has been followed during product development. However, this is often not the case in product development due to time and financial constraints, as well as the fact that probiotic products are likely to have several mechanisms of action and effectors (Lebeer et al., 2018), making it particularly challenging to fully control development and production process. Nevertheless, specific theoretical modeling approaches can be made especially with the traditional well-studied probiotic strains where the metabolism is well understood and relatively easy to follow and model, Box 1. Similarly, the possible combination of probiotics with other substances (e.g., vitamins, prebiotics, polyphenols, minerals, etc.) or additional probiotic strains may need to be tested at the preclinical stage for compatibility and stability. Potential synergies may be modeled using the above discussed metabolic mapping, as mentioned in Box 1.

Importantly, the time and effort necessary to develop a novel probiotic product can differ significantly due to legislation, health claims, variable costs, market value and other parameters in each respective PF, PDS, MF/FSMP, or LBPs category (Table 1). Consequently, in addition to regulatory considerations and intended product use, development costs and timing can be a decisive factor for the probiotic product category that the manufacturer will ultimately target. Box 1 outlines the points that are key in the optimal product development and have a strong impact on the price of the development and the length of the process.

In the end, successful product development stands or falls with the stability (i.e., survival) of the probiotic in the product until the end of shelf life. Here, two factors are of main importance: moisture and temperature. Except for high moisture probiotic foods such as yogurts and juices, probiotics retain their viability best at low moisture (such as infant formula powders, capsules, sachets, etc.). For all probiotic products low temperatures are preferred to improve or maintain stability. Appropriate storage conditions should therefore be noted on the label. Notwithstanding all precautions, viability of probiotics tends to decline over time (Forssten et al., 2011). By studying strain stability, the decline under given storage conditions is known. It is common industry practice to apply an overage to compensate for this loss and assure the intended dose is still present at the end of shelf life. In this way the product provides the ‘adequate amount’ as stipulated in the probiotic definition (Fusco et al., 2022).

To assure consumers that they purchase the product they think, it is essential that proper declaration of the included probiotic strains are given on the label of the product (Fusco et al., 2022). These include:

• Ingredients/allergens: all active and inert ingredients are listed in descending order by weight (it is mandatory for any allergens to be included in this list).
• Genus, species, (subspecies) and strain of the probiotic, according to current taxonomy.
• Viable count in colony forming units (CFUs), as total count, although counts for each strain are preferred. CFUs listed on the label should at least be equal to the amount shown to be efficacious in human studies and should be guaranteed until end of shelf life.
• Daily dosage: the amount that needs to be consumed daily, which should be at or above the daily dose tested in human studies.
• Claim/recommended use (if allowed by the local/regional regulator): it provides information about how to use the product and what benefit can be expected from the product. Any claim must be scientifically substantiated and evaluated and approved by a national or international regulator.
• Storage information: how to store the product to maintain the probiotic potency.
• Best before date: provides information on how long the probiotic product will contain adequate amount of probiotics to deliver any claimed benefit.
• Company name/contact information: consumers should always be able to contact the company with questions to get more information or to report any adverse effect.

## 5 Clinical research strategies

Clinical research comprising human intervention studies is key to demonstrating the efficacy of a probiotic product to either maintain human health (PF, PDS) or to mitigate, prevent or cure diseases (LBPs) in the target population, and thus to support specific health claims (EFSA Panel on Dietetic Products, Nutrition and Allergies, 2016; United States Food and Drug Administration [FDA], 2016, 2022a; Paquet et al., 2021). The regulations, design, evaluation criteria and costs of clinical trials for PF and PDS versus LBPs reflect the differences in intended use of these probiotic categories. Detailed information on the similarities and differences in designing, executing, and interpreting a clinical trial for PF, PDS, and LBPs is provided in Table 2, with the following section highlighting several key points.

Next to safety testing, a screening for functional features allows the establishment of potential beneficial action(s) of a probiotic product and, consequently, its potential as either PF/PDS or LBPs. Such preclinical testing can comprise testing for anti-pathogenic action, immunomodulatory interactions with host cells, mucosal epithelial barrier enhancement and/or production of bioactive molecules. Preclinical characterization of a strain’s mechanisms of action is not a strict requirement, and in fact cannot be sufficient on its own to designate a microorganism as “probiotic” (Binda et al., 2020). It can help in designing the clinical study and choosing potential biomarkers of health and disease related to the expected mechanisms of action. This underlines the translational importance of mode of action research translating forward to regulatory category and target market (Kleerebezem et al., 2019). Of note, pre-clinical studies do not necessarily predict the exact mechanism of action in clinical settings due to intrinsic differences between *in vitro*, *ex vivo* and animal models compared to humans.

The required levels of clinical evidence to support a particular probiotic product will depend on the envisioned probiotic category. Randomized, double-blind, placebo-controlled trials provide the strongest clinical evidence to establish causal relationships between probiotic product intake and health outcomes required by the authorities (Shane et al., 2010). A recent review reported more than 1,000 clinical trials on probiotic products registered on ClinicalTrials.gov and/or the International Clinical Trials Registry Platform, with most studies conducted in the USA or Europe and averaging 74 participants per trial (Dronkers et al., 2020). Of these studies, 43% included healthy volunteers, this in contrast to registered clinical trials in general with 25% enrolled healthy participants. This is not surprising, as the beneficial effects of PF/PDS must be demonstrated in healthy populations (EFSA Panel on Dietetic Products, Nutrition and Allergies, 2016).

However, such studies remain challenging, especially for the PF and PDS categories. Appropriate new clinical outcome measures/biomarkers specifically affected by probiotics are not always known, which can hamper probiotic claims, for example in relation to (immune) defenses against pathogens or support of normal bodily functions (EFSA Panel on Dietetic Products, Nutrition and Allergies, 2016). Mechanisms of action are typically multi-faceted and the magnitude of effect which can be observed when administering PF and PDS in a healthy population can be subtle compared to diseased populations. When designing a clinical study for a product in the PF/PDS category, a validated biomarker demonstrating reduced risk reduction in a healthy population (e.g., cholesterol levels for heart disease or postprandial glucose levels for pre-diabetes) would be sufficient (Quigley, 2022). However, such biomarkers of risk reduction are minimally established (Table 2) and can be highly challenging to identify for specific health outcomes such as irritable bowel syndrome (IBS) (Quigley, 2022). Furthermore, large study cohorts might be required to observe sufficient disease risk reduction. Biomarkers of disease are often easier to detect, though their use implies that the clinical effects of the probiotic product are rather explored in the drug category to treat, prevent, mitigate or cure a condition in a diseased population (Shane et al., 2010). Consequently, when making a disease-modifying claim for a LBPs, the standards for drug claims must be met; typically including a phase III clinical trial.

Clinical research of PF, PDS, but especially LBPs, is linked to selective pressures relating to stakeholder involvement in research and product development, as extensively described in Table 2. In this perspective the FDA regulations state that the early preclinical development of an investigational new drug by the drug’s sponsor (manufacturer or potential marketer) has the aim to determine the safety and pharmacological activity before proceeding to early-stage clinical studies (United States Food and Drug Administration [FDA], 2022a,b). The pre-human phase (time from synthesis to clinical testing) for drugs is expensive and time-consuming, as it has been reported to average a considerable 31.7 months with inferred out-of-pocket costs of 430 million USD based on 1990–2013 data on 106 new drugs from 10 biopharmaceutical companies (DiMasi et al., 2016). In a new development, a recent law in the USA has eliminated the requirement to test novel drugs or biologics in animal models before proceeding to clinical trials in humans, allowing to replace the more laborious and costly animal testing by cell-based assays, computer modeling or “organs on a chip” as potential alternatives [Section 3209 “Animal Testing Alternatives” of the appropriations bill amending section 505 of the 21 U.S.C. 355 (H.R.2617 - 117th Congress, 2022)]. However, companies still need to present enough data to convince the FDA, which can be challenging, as alternative *in vitro* and *ex vivo* methods are not always entirely representative of complex *in vivo* interactions. These alternatives are already implemented in PF and PDS probiotic research. In any case, in contrast to PF and PDS, more extensive information on pharmacology and toxicology studies, manufacturing information, and clinical protocols and investigator information are required for FDA revision and initiation of clinical studies to test the therapeutic potential of a new drug (United States Food and Drug Administration [FDA], 2022b). When considering all drug types, it has been reported that ultimately only 10.4% of drug indication development paths in phase 1 achieved FDA approval (Hay et al., 2014). Considering the biological complexity of LBPs, these numbers can be expected to be even lower, which represents an issue for stakeholders, including drug sponsors, developers, investors, and the target patient population.

Following clinical trials, the results on the LBPs quality, safety and efficacy are used by the competent authorities (e.g., FDA or EMA) to assess the benefit–risk balance (or burden to benefit ratio) as a key parameter of drug evaluation. Furthermore, potency testing to evaluate and measure the drug efficacy is another key parameter distinguishing the LBPs category from PF/PDS (where is it not applied) and is used for the drug manufacturing parameters. On the other hand, an important parameter for the PF/PDS category versus the LBPs category is the history of safe use in food products. Strains that do not belong to species with such history of safe use can be included in PF/PDS as well but may need to comply with additional safety testing (Roe et al., 2022). When compared to drugs, the average severity of adverse effects (AE) and serious adverse effects (SAEs) in PF/PDS and LBPs clinical studies is minimal and their incidence is very low and rare, respectively (Table 2). Nevertheless, AE should be monitored and reported in all probiotic clinical trials, as the potential severity of side effects can still range from low to very high at the extremes, while there is no acceptance of side effects for PF/PDS. AE should also not be confused with product tolerability; the latter is a more common occurrence for PF/PDS especially in gastrointestinal discomfort. A recent analysis highlighted inadequate and incomplete adverse events/serious adverse events/biosurveillance reporting in the context of probiotic interventions in randomized clinical trials in IBS compared to drug interventions, which hampers the assessment of burden to benefit ratio (van der Geest et al., 2022). To implement AE monitoring in practice, an extension of the CONSORT statement provides guidelines for evaluation of AE applicable to all clinical research (Ioannidis et al., 2004). To monitor and classify AE according to their severity, official guidelines such as Common Terminology Criteria for Adverse Events (CTCAE) established by the Department of Health and Human Services (USA) can be used.

While no reimbursement schemes are currently available for PF and PDS, several modeling studies have highlighted the economic benefits with the use of probiotic products by the general population. For example, probiotic use in the USA could reduce the 2017–2018 economic burden of flu-like respiratory tract infections for the health care payer by 4.6 million–373 million USD according to a state-transition microsimulation model implemented by Lenoir-Wijnkoop et al. (2019). Similar estimates have been made for France and Canada (Lenoir-Wijnkoop et al., 2015, 2016). Real-time data obtained in studies in various populations support the results of the modeled studies (Leal et al., 2016; Shen et al., 2017; Lau et al., 2020). Reimbursement could be possible for LBPs according to general drug regulation, however it should be noted that for a drug to be considered for reimbursement in the USA or EU it needs to demonstrate clinical benefits and a lower risk profile compared to the established standard of care (HealthEcon, 2023). It is crucial to consider the evaluation guidelines implemented by the national Health Technology Assessments (HTA) agencies during clinical trial planning to include the relevant endpoints necessary for a positive reimbursement evaluation.

## 6 Future perspectives and conclusion

The number of probiotic products on the market and their value has increased over the years; this was especially true during the COVID-19 pandemic (Lumina Intelligence, 2021). Since then, some markets may have shown signs of slowing down which may also be driven by economic circumstances. Nevertheless, all three probiotic product categories (PF, PDS, or LBPs) are promising. However, careful consideration of which category is to be targeted is required, as this will dictate the applicable regulations, tailored clinical trial design, product development and monitoring strategy, production and all the associated costs. Expectations should be managed when understanding how probiotic products perform within each category (physiologically, commercially, etc.), especially on the level of efficacy and related parameters. Each product category is uniquely situated within its scope of intended use, and as such their intrinsic differences will lend themselves to potentially unwarranted comparison which contributes to confusion.

The probiotic regulatory framework is rapidly evolving, especially for the LBPs category, which offers both novel possibilities and specific challenges in meeting potency and stability requirements that are crucial both for the regulatory authorities and consumers. Of note, the longer clinical development of LBPs may result in markedly higher development costs, ultimately to be borne by final customers in the form of higher product prices. Further, LBPs maybe so-called NGPs (Supplementary Table 1) which may have specific growth and stability challenges; further increasing cost. Thus, it is important for such LBPs to demonstrate a significant therapeutic gain compared to general PF/PDS to warrant the extra cost, Tables 1, 2. Which is also to be expected as LBPs, by definition, are drugs and thus aimed at treating, preventing and mitigating disease (Gulliver et al., 2022).

On the other hand, for PF/PDS seeking a health claim, the requirement to target healthy populations may necessitate large cohorts in order to detect minute changes in biomarkers (changes in biomarkers tend to be stymied in healthy populations due to homeostasis). This will drive development costs and product prices up. It remains to be seen if customers will be willing to pay for such health claims in sufficient numbers to produce a meaningful return on investment. The consumer will compare the price of a PF/PDS with its standard counterpart and may not be willing or able to pay the difference in cost, Tables 1, 2. In healthy subjects probiotic consumption, whether as PF, PDS or LBP, generally does not lead to changes in the fecal microbiota composition, although this may be different for patients (Sanders, 2016). To better understand this and what effect large scale consumption of probiotics has on the fecal microbiota composition appropriate studies should be performed.

Further, significant differences in regulation still exist between geographical regions and countries, even within the same country or region. This can be seen in the EU where countries such as, e.g., France Italy and Spain allow the use of the term ‘probiotic’ as a category while this is not allowed in, e.g., Germany, Finland and Sweden. Between jurisdictions, the EU seems to have the narrowest interpretations for health claims approvals, while for instance Canada and Brazil allow for defined generic and science-based specific claims, provided they are supported by adequate evidence without the need to a pre-marketing approval. US regulation allows for so-called structure function claims. However, strong health claims in general do always require premarket approval by the regulators. Certain markets are currently more promising to target commercially than others regarding formulation, labeling and health claims.

In summary, all three probiotic categories are subject to product regulation. Whether they require a pre-marketing registration for the finished product or the ingredient, a notification, have immediate access to the market, or are part of established positive or negative lists, whether claims about their benefits can be made or not, whether the evidence is to be submitted to the authorities or not, a probiotic must be characterized, safe, produced following controlled quality, meet its specifications, lawfully marketed, and adequately labeled according to the intended use with truthful messages that are supported by verifiable data. Safety remains the bare minimum key factor, and this does not stop at the market access step. Monitoring adverse events and reporting serious ones is a general requirement that must be performed, which will increase knowledge and trust, and keep the door open for future perspectives and opportunities.

## Author contributions

IS: Conceptualization, Project administration, Visualization, Writing—original draft, Writing—review and editing. SB: Conceptualization, Writing—original draft, Writing—review and editing. JH: Conceptualization, Writing—original draft, Writing—review and editing. SH: Conceptualization, Writing—original draft, Writing—review and editing. SL-R: Conceptualization, Writing—original draft, Writing—review and editing. JD: Writing—review and editing. JE-M: Writing—review and editing. PL: Writing—review and editing. RM: Writing—review and editing. MP: Writing—review and editing. AO: Conceptualization, Project administration, Writing—original draft, Writing—review and editing.
