# Supplementary material for: Comparing technology and regulatory landscape of probiotics as food, dietary supplements and live biotherapeutics
Source: Front Microbiol. 2023 Dec 19;14:1272754. doi: 10.3389/fmicb.2023.1272754 (PMC10770255; doi:10.3389/fmicb.2023.1272754)
Supplement: Supplementary file 1 [file Table_1.docx]

**Supplementary Table 1** Relevant examples of Live Biotherapeutics in different geographies.

| **Type of probiotic product** | **Product and/or strain name** | **Geographical region** | **Reference** |
| --- | --- | --- | --- |
| LBP for treatment and prevention of diarrhea | *Enterococcus faecium* SF68 | [Marketed/licensed](https://cerbios.swiss/product/new-flora-probiotics-sf68/) in Austria, Italy, Switzerland, Pakistan, Korea, Singapore; registered in Mexico and Canada | (Holzapfel et al., 2018) |
| LBP against bacterial vaginosis | LACTIN-V containing *Lactobacillus crispatus* CTV-05 | [Awaiting](https://oselinc.com/frequently-asked-questions-2/) Phase 3 clinical development and marketing authorization by the FDA in the USA | (Armstrong et al., 2022) |
| LBP against *Clostridium difficile* infection | Vowst (live-brpk, formerly SER-109) with fecal bacterial spores from healthy volunteers | [Approved](https://www.fda.gov/news-events/press-announcements/fda-approves-first-orally-administered-fecal-microbiota-product-prevention-recurrence-clostridioides) for medical use in the USA | (Khanna et al., 2022) |
| LBP against *Clostridium difficile* infection | VE303 with 8 commensal clostridial strains of human origin | [Fast Track](https://www.businesswire.com/news/home/20230508005499/en/PureTech-Founded-Entity-Vedanta-Biosciences-Receives-Fast-Track-Designation-for-VE303-and-Presents-Phase-2-Data-at-Digestive-Disease-Week) designation to expedite the review of VE303 by FDA in the USA | (Louie et al., 2023) |
| LBP against *Clostridioides difficile* infection | Non-toxigenic *C.difficile* strain M3 (NTCD-M3) | [Awaiting](https://oselinc.com/frequently-asked-questions-2/) Phase 3 clinical development and marketing authorization by the FDA in the USA | (Sambol et al., 2023) |
| LBP against irritable bowel syndrome | Blautix (MRx1234) with *Blautia hydrogenotrophica* | Completed a successful Phase II trial, but not yet on the market | (Quigley et al., 2023) |
| LBP against obesity and metabolic syndrome | Xla1 containing *Christensenella minuta* | Granted Investigational New Drug (IND) authorization by the FDA in the USA; scientific advice from EMA | (Paquet et al., 2021) |
| LBP against ulcerative colitis | Mutaflor® containing *Escherichia coli* Nissle 1917 | Marketed in Germany, Canada, Singapore, Australia, and New Zealand; [not available](https://www.regulations.gov/document/FDA-2012-S-1178-0014) in the USA | (Rembacken et al., 1999) |
| LBP consortia against ulcerative colitis | MH002 consortia of 6 commensal strains | Phase Ib/IIa study in patients with mild-to-moderate UC | <https://www.clinicaltrialsregister.eu/ctr-search/search?query=2020-004355-33> |
| LBP consortia against pouchitis | MH002 consortia of 6 commensal strains | Phase II trial ongoing | <https://www.clinicaltrialsregister.eu/ctr-search/search?query=2021-006656-14> |
| LBP against psoriasis | EDP1815 containing *Prevotella histicola* | Phase II trial ongoing | <https://classic.clinicaltrials.gov/ct2/show/NCT04603027> |

References:

Armstrong, E., Hemmerling, A., Miller, S., Burke, K. E., Newmann, S. J., Morris, S. R., et al. (2022). Sustained effect of LACTIN-V (Lactobacillus crispatus CTV-05) on genital immunology following standard bacterial vaginosis treatment: results from a randomised, placebo-controlled trial. Lancet Microbe 3, e435–e442.

Holzapfel, W., A. Arini, M. Aeschbacher, R. Coppolecchia and B. Pot. 2018. Enterococcus faecium SF68 as a model for efficacy and safety evaluation of pharmaceutical probiotics. Beneficial Microbes 9(3): 375-388.

Khanna, S., Sims, M., Louie, T. J., Fischer, M., LaPlante, K., Allegretti, J., et al. (2022). SER-109: an oral investigational microbiome therapeutic for patients with Recurrent Clostridioides difficile Infection (rCDI). Antibiotics 11:1234. doi: 10.3390/ antibiotics11091234

Louie, T., Golan, Y., Khanna, S., Bobilev, D., Erpelding, N., Fratazzi, C., et al. (2023). VE303, a defined bacterial consortium, for prevention of recurrent Clostridioides difficile infection: a randomized clinical trial. JAMA 329, 1356–1366.

Paquet, J.-C., Claus, S. P., Cordaillat-Simmons, M., Mazier, W., Rawadi, G., Rinaldi, L., et al. (2021). Entering first-in-human clinical study with a single-strain live biotherapeutic product: input and feedback gained from the EMA and the FDA. Front. Med. 8:716266. doi: 10.3389/fmed.2021.716266

Quigley, E. M. M., Markinson, L., Stevenson, A., Treasure, F. P., and Lacy, B. E. (2023). Randomised clinical trial: efficacy and safety of the live biotherapeutic product MRx1234 in patients with irritable bowel syndrome. Aliment. Pharmacol. Ther. 57, 81–93. doi: 10.1111/apt.17310

Rembacken, B. J., Snelling, A. M., Hawkey, P. M., Chalmers, D. M., and Axon, A. T. R. (1999). Non-pathogenic Escherichia coli versus mesalazine for the treatment of ulcerative colitis: a randomised trial. Lancet 354, 635–639. doi: 10.1016/S0140-6736(98)06343-0

Sambol, S. P., Skinner, A. M., Serna-Perez, F., Owen, B., Gerding, D. N., and Johnson, S. (2023). Effective colonization by nontoxigenic Clostridioides difficile REA strain M3 (NTCD-M3) spores following treatment with either fidaxomicin or vancomycin. Microbiol. Spectr. 11, e517–e523. doi: 10.1128/spectrum.00517-23
